# Corticosteroid-induced intraocular pressure elevation in the pediatric patients: A protocol for systematic review

**DOI:** 10.1097/MD.0000000000028189

**Published:** 2021-12-10

**Authors:** Guangjun Xu, Jiaoni Zheng, Jianghui Cai, Jing Zhang, Jue Liu, Shu Tang

**Affiliations:** a Department of Ophthalmology, Chongqing University Center Hospital, Chongqing Emergency Medical Center, Chongqing Fourth People's Hospital, Chongqing, China; b Department of Pharmacy, Chongqing University Center Hospital, Chongqing Emergency Medical Center, Chongqing Fourth People's Hospital, Chongqing, China; c Department of Pharmacy, Chengdu Women's and Children's Central Hospital, School of Medicine, University of Electronic Science and Technology of China, Chengdu, China; d Department of Physical Examination Center, The Second Affiliated Hospital of Chongqing Medical University, Chongqing, China.

**Keywords:** corticosteroid, intraocular pressure, pediatric, protocol

## Abstract

**Background:**

Corticosteroids have been one of the most frequently used therapeutics in ophthalmology over the past decades, known for their potent anti-inflammatory and immunosuppressive actions. Intraocular pressure elevation has proven to be a significant ocular side effect that could accompany steroid use. However, the information on ocular-hypertensive corticosteroid response is scant in children. We aim to systematically describe the corticosteroid-induced intraocular pressure elevation in the pediatric age group.

**Methods:**

PubMed, Embase, Web of Science, Cochrane Library, Latin American and Caribbean Health Sciences Literature, and the Chinese Biomedical Literature database will be searched for potential articles from database inception to April 29, 2021. No language restrictions will be applied. Studies involving patients less than 18 years old receiving corticosteroids will be included. We will screen abstracts for relevance, extract data, and assess the risk of bias in duplicate. We will rate the certainty of evidence using the Grading of Recommendations Assessment Development and Evaluation approach. The primary outcome will be the intraocular pressure in pediatric patients group. We will provide a narrative synthesis of the findings.

**Results:**

The systematic review will provide high-quality evidence to assess the relationship between dosage, frequency, route of administration, and duration of corticosteroid on intraocular pressure in children.

**Conclusion:**

The systematic review will provide evidence to assess the safety of corticosteroid for ocular diseases in pediatric population.

**PROSPERO registration number:**

CRD42021252298.

## 1 Introduction

Corticosteroids have been one of the most widely used therapeutics in treating various ocular diseases for many years, known for their potent anti-inflammatory and immunosuppressive actions.^[1]^ Corticosteroids can exert various side effects.^[2]^ Intraocular pressure (IOP) elevation has proven to be one of their major side effects.^[3,4]^ Nevertheless, corticosteroids in different dosage forms are indispensable in treating inflammatory eye diseases. In addition, corticosteroids are the mainstay of treatment in postoperative ocular management. It prevents or suppresses the undesirable consequences of postoperative ocular inflammation, including redness, swelling, and tenderness.

The ocular-hypertensive corticosteroid response in adults to oral,^[5]^ topical dermatologic,^[6]^ topical ocular,^[7]^ intravenous^[8]^ has been well documented. However, compared with adults, the literature on corticosteroid therapy's effect on IOP in the pediatric population is limited and controversial.^[9,10]^ Moreover, the relationship between dosage, frequency, route of administration, and duration of corticosteroid on IOP in children remains unclear. Thus, there is a need to qualitatively and critically appraise available evidence and evaluate where gaps exist.

## 2 Objectives

Based on the above considerations, this systematic review protocol aims to evaluate corticosteroid-induced IOP elevation in the pediatric population.

## 3 Methods

This protocol conforms to the preferred reporting items for systematic review and meta-analysis protocols.^[11]^ This systematic review protocol was registered with the International Prospective Register of Systematic Reviews (PROSPERO) (registration number CRD42021252298).

### 3.1 Eligibility criteria

Studies will be selected according to the criteria outlined below.

### 3.2 Study designs

We will include randomized controlled studies or observational studies (including cohort, case-control studies, case series, and case reports) involving patients under 18 years old receiving corticosteroids. Studies with available clinical characteristics, including ocular side effects of corticosteroids will be included. We will exclude data from unpublished reports, reviews, guidelines, opinions, editorials, letters, comments, and nonhuman studies. Duplicate or substudy of previously published investigations will be removed.

### 3.3 Participants

This review will include studies conducted among pediatric patients (<18 years old) receiving corticosteroids.

### 3.4 Intervention

In this review, receiving corticosteroids will be considered as exposures/interventions. There will be no restrictions based on dosing, timing, frequency, route of administration or therapy duration.

### 3.5 Comparators

The comparator could be no treatment, placebo or other treatments excluding corticosteroids.

### 3.6 Outcomes

The primary outcome will be the intraocular pressure in pediatric population.

### 3.7 Setting, language, and timing

There will be no restrictions on geographic location, setting, language or publication years when selecting studies.

### 3.8 Information sources and search strategy

We will conduct a comprehensive literature search of PubMed, Embase, Web of Science, the Cochrane Library, LILACS, and the Chinese Biomedical Literature Database (CBM) from database inception to April 29, 2021. The reference lists of the relevant articles will also be searched. We do not impose any language restrictions. Combinations of the following keywords and Medical Subject Headings terms (MeSH) terms will be used: corticosteroid, corticosteroids, corticosteroidal, corticosteroide, adrenal cortex hormones, steroid, intraocular pressure, intraocular, pressure, Ocular hypertension, Ocular hypertensive, OHT, paediatrics, paediatric, pediatrics, pediatric, child, children, baby, toddler, infant, infants, newborn, newborns, neonate, neonates, adolescent. The proposed full search strategy combining free text and MeSH terms for all databases is outlined in Appendix 1, Supplemental Digital Content.

### 3.9 Study records

#### 3.9.1 Data management and selection process

Following the search, duplicate articles will be identified and removed using EndNote software version X7 (Clarivate Analytics). Two reviewers (JC and JZ) will independently evaluate the search articles for potential inclusion by screening titles and abstracts. Two other authors (JL and ST) will assess the full texts of those identified as relevant to determine eligibility for final inclusion. The results will be discussed between each assessment to reach a consensus on the interpretation of the inclusion criteria. Any disagreements regarding study eligibility will be resolved by consensus, and a third reviewer (JZ) will be consulted, if necessary. We will document the excluded studies and reasons for exclusion, the selection of studies will be summarized in a PRISMA flowchart.

Study selection will be managed using Covidence software (www.covidence.org). To ensure consistency across reviewers, we will conduct calibration exercises before starting the review. Data will be extracted from included studies by 2 independent authors (GX and JZ). If the information required to assess eligibility is unavailable or unclear, we will attempt to contact the authors of the original reports and will ask them to provide further details.

#### 3.9.2 Data extraction and data items

The identified publication(s) will be analyzed using criteria based on the maximum correspondence with the inclusion criteria and minimal risk of bias. When a hospital had published their cases more than once, if the recruitment periods overlapped, only the most informative study with the bigger sample size was included to minimize the possibility of double counting.^[12]^

To minimize errors, a data extraction form has been developed with consensus among the authors. Data will then be extracted from the studies selected for inclusion, as follows:

- General characteristics of the study (author names, title, recruitment periods, and publication date);
- Type of the study;
- Sample size;
- Study subject characteristics (demographic characteristics, dosage, routes of corticosteroids administration, definition of IOP, and ethnic group);
- Outcome measures and analyses (intraocular pressure);
- Study findings.

### 3.10 Risk of bias assessment and GRADE certainty assessment

We will use the Cochrane Risk of Bias tool 2.0 (RoB 2.0) for Randomized controlled studies.^[13]^ The RoB 2.0 assessment has 5 domains, and 1 overall risk of bias domain, The risk of bias will be assessed as “low”, “some concern” or “high” in each domain. Two reviewers (JL and ST) will independently evaluate the risk of bias using the Newcastle-Ottawa scale (NOS) for observational cohort and case-control studies and Joanna Briggs Institute (JBI) critical appraisal tools for case reports and case series studies.^[14,15]^ For cohort and case-control studies, there are 3 grouping items as follows: selection, comparability, exposure/outcomes. A study can be awarded a maximum of 1 star for each numbered item within the selection and outcome categories. A maximum of 2 stars can be given for comparability. More stars equalling lower risk. Case reports and case series studies will be categorized according to the percentage of positive answers to each question. Low risk of bias indicated more than 70% of positive responses; moderate risk of bias ranged between 50% and 69%, and high risk of bias represented less than 49% of positive answers. Any disagreements that arise between the reviewers will be resolved through discussion. A third reviewer (JZ) will settle unresolved disputes. The results of the critical appraisal will be reported in narrative form and in a table. We will grade the certainty of evidence using the GRADE approach. We will use the GRADEpro guideline development tool (GDT) app to rate evidence and present it in a summary of findings table.

### 3.11 Data synthesis

All studies fulfilling the eligibility criteria will be included in the qualitative synthesis. We will provide a narrative synthesis of the findings when meta-analysis is impossible or inappropriate from the included studies, structured around the study design, the target population characteristics, the follow-up period (if applicable), the type of outcome(s), intraocular pressure in children receiving corticosteroids. We will provide information presented in the text and tables to summarize and explain the characteristics and findings of the included studies.

Additional analyses will be performed if applicable, as follow:

- We will conduct subgroup analyses by patients who are ≤9 years of age or >10 years of age;
- We will conduct subgroup analyses by different routes of corticosteroids administration;
- We will conduct subgroup analyses by the dosage of corticosteroids;
- We will conduct subgroup analyses by different kinds of corticosteroids.

## 4 Ethics and dissemination

This study does not require ethical approval. The findings of this systematic review will be published in a peer-reviewed journal.

## Author contributions

JC, JL, and ST are the guarantors. GX, JZ, and JC drafted the manuscript. JL contributed to the design of the search strategy. JZ provided expertise on intraocular pressure elevation. All authors contributed to the development of the selection criteria, the risk of bias assessment strategy and data extraction criteria. All authors read, provided feedback and approved the final version.

**Investigation:** Jing Zhang.

**Supervision:** Jue Liu, Shu Tang.

**Validation:** Jue Liu, Shu Tang.

**Writing – original draft:** Guangjun Xu, Jiaoni Zheng, Jianghui Cai.

## Supplementary Material

Supplemental Digital Content
