# Supplementary material for: Corticosteroid-induced intraocular pressure elevation in the pediatric patients: A protocol for systematic review
Source: Medicine (Baltimore). 2021 Dec 10;100(49):e28189. doi: 10.1097/MD.0000000000028189 (PMC8663802; doi:10.1097/MD.0000000000028189)
Supplement: Supplemental Digital Content [file medi-100-e28189-s001.doc]

**Appendix 1: Search strategies.**

**PubMed**

**Search** **Query**

#1 Search: (Adrenal Cortex Hormones[mh]) OR (Hormones, Adrenal Cortex[Title/Abstract]) OR (Adrenal Cortex Hormone[Title/Abstract]) OR (Cortex Hormone, Adrenal[Title/Abstract]) OR (Hormone, Adrenal Cortex[Title/Abstract]) OR (Corticosteroids[Title/Abstract]) OR (Corticosteroid[Title/Abstract]) OR (Corticoids[Title/Abstract]) OR (Corticoid[Title/Abstract])

#2 Search: (Dexamethasone[mh]) OR (Methylfluorprednisolone[Title/Abstract]) OR (Hexadecadrol[Title/Abstract]) OR (Decameth[Title/Abstract]) OR (Decaspray[Title/Abstract]) OR (Dexasone[Title/Abstract]) OR (Dexpak[Title/Abstract]) OR (Maxidex[Title/Abstract]) OR (Millicorten[Title/Abstract]) OR (Oradexon[Title/Abstract]) OR (Decaject[Title/Abstract]) OR (Decaject-L.A.[Title/Abstract]) OR (Decaject L.A.[Title/Abstract]) OR (Hexadrol[Title/Abstract])

#3 Search: (Intraocular Pressure[mh]) OR (Intraocular Pressures[Title/Abstract]) OR (Pressure, Intraocular[Title/Abstract]) OR (Pressures, Intraocular[Title/Abstract]) OR (Ocular Tension[Title/Abstract]) OR (Ocular Tensions[Title/Abstract]) OR (Tension, Ocular[Title/Abstract]) OR (Tensions, Ocular[Title/Abstract]) OR (Ocular hypertension[Title/Abstract]) OR (OHT[Title/Abstract])

#4 Search: (paediatrics[Title/Abstract]) OR (paediatric[Title/Abstract]) OR (pediatrics[Title/Abstract]) OR (pediatric[Title/Abstract]) OR (child[Title/Abstract]) OR (children[Title/Abstract]) OR (baby[Title/Abstract]) OR (toddler[Title/Abstract]) (infant[Title/Abstract]) OR (infants[Title/Abstract]) OR (newborns[Title/Abstract]) OR (newborn[Title/Abstract]) OR (neonate[Title/Abstract]) OR (neonates[Title/Abstract]) OR (adolescent[Title/Abstract])

#5 Search: #1 or #2

#6 Search: #3 and #4 and #5

**Embase**

**No.** **Query**

#1 'Intraocular Pressure'/ab,ti

#2 'Corticosteroid' or 'Dexamethasone'/ab,ti

#3 'pediatric' or 'child' or 'adolescent'/ab,ti

#4 #1 and #2 and #3

**Cochrane Library**

**ID Search**

#1 (Dexamethasone):ti,ab,kw (Word variations have been searched)

#2 ("Corticosteroid"):ti,ab,kw (Word variations have been searched)

#3 ("intraocular pressure"):ti,ab,kw (Word variations have been searched)

#4 (pediatric or paediatric or baby or child or children or adolescent or infant or newborn or neonate):ti,ab,kw (Word variations have been searched)

#5 #1 OR #2

#6 #3 AND # 4 AND #5

**Web of Science** (Index=SCI-EXPANDED, SSCI, A&HCI, ESCI)

# 18 #9 AND #16 AND #17

# 17 #13 OR #14 OR #15

# 16 #10 OR #11 OR #12

# 15 KP=(paediatrics OR paediatric OR pediatrics OR pediatric OR child OR children OR baby OR toddler infant OR infants OR newborns OR newborn OR neonate OR neonates OR adolescent)

# 14 TI=(paediatrics OR paediatric OR pediatrics OR pediatric OR child OR children OR baby OR toddler infant OR infants OR newborns OR newborn OR neonate OR neonates OR adolescent)

# 13 AB=(paediatrics OR paediatric OR pediatrics OR pediatric OR child OR children OR baby OR toddler infant OR infants OR newborns OR newborn OR neonate OR neonates OR adolescent)

# 12 KP=(Intraocular Pressure OR Intraocular Pressures OR Pressure, Intraocular OR Pressures, Intraocular OR Ocular Tension OR Ocular Tensions OR Tension, Ocular OR Tensions, Ocular OR Ocular hypertension OR OHT)

# 11 TI=(Intraocular Pressure OR Intraocular Pressures OR Pressure, Intraocular OR Pressures, Intraocular OR Ocular Tension OR Ocular Tensions OR Tension, Ocular OR Tensions, Ocular OR Ocular hypertension OR OHT)

# 10 AB=(Intraocular Pressure OR Intraocular Pressures OR Pressure, Intraocular OR Pressures, Intraocular OR Ocular Tension OR Ocular Tensions OR Tension, Ocular OR Tensions, Ocular OR Ocular hypertension OR OHT)

# 9 #4 OR #8

# 8 #7 OR #6 OR #5

# 7 KP=(Dexamethasone OR Methylfluorprednisolone OR Hexadecadrol OR Decameth OR Decaspray OR Dexasone OR Dexpak OR Maxidex OR Millicorten OR Oradexon OR Decaject OR Decaject-L.A.OR Decaject L.A. OR Hexadrol)

# 6 TI=(Dexamethasone OR Methylfluorprednisolone OR Hexadecadrol OR Decameth OR Decaspray OR Dexasone OR Dexpak OR Maxidex OR Millicorten OR Oradexon OR Decaject OR Decaject-L.A.OR Decaject L.A. OR Hexadrol)

# 5 AB=(Dexamethasone OR Methylfluorprednisolone OR Hexadecadrol OR Decameth OR Decaspray OR Dexasone OR Dexpak OR Maxidex OR Millicorten OR Oradexon OR Decaject OR Decaject-L.A.OR Decaject L.A. OR Hexadrol)

# 4 #3 OR #2 OR #1

# 3 KP=( Adrenal Cortex Hormones OR Hormones, Adrenal Cortex OR Adrenal Cortex Hormone OR Cortex Hormone, Adrenal OR Hormone, Adrenal Cortex OR Corticosteroids OR Corticosteroid OR Corticoids OR Corticoid)

# 2 TI=( Adrenal Cortex Hormones OR Hormones, Adrenal Cortex OR Adrenal Cortex Hormone OR Cortex Hormone, Adrenal OR Hormone, Adrenal Cortex OR Corticosteroids OR Corticosteroid OR Corticoids OR Corticoid)

# 1 AB=( Adrenal Cortex Hormones OR Hormones, Adrenal Cortex OR Adrenal Cortex Hormone OR Cortex Hormone, Adrenal OR Hormone, Adrenal Cortex OR Corticosteroids OR Corticosteroid OR Corticoids OR Corticoid)

**Latin American and Caribbean Health Sciences Literature (LILACS)**

**No Search in the filed**

1 Intraocular Pressure [Words]

2 child or paediatric or pediatric [Words]

3 Corticosteroid or Dexamethasone [Words]

**Chinese Biomedical Literature database (CBM) - field searching in Chinese**

("眼压"[常用字段:智能] And "儿童"[常用字段:智能] And "激素"[常用字段:智能]) AND 1981-2021[日期]
